# Evolving Trends in the Management of Acute Appendicitis During COVID-19 Waves: The ACIE Appy II Study

**DOI:** 10.1007/s00268-022-06649-z

**Published:** 2022-07-09

**Authors:** Francesco Pata, Marcello Di Martino, Mauro Podda, Salomone Di Saverio, Benedetto Ielpo, Gianluca Pellino, the ACIE Appy Study Collaborative, Abba Julio, Abdullah Alshamrani, Abdullah Alturkistani, Abdulrahman Alghamdi, Abdulrahman Almalki, Adam Orengia, Adnan Kuvvetli, Adolfo Pisanu, Adrian Smith, Adriana Michelle Treviño Figueroa, Aeris Jane Nacion, Ahmad Alhazmi, Ahmad Bouhuwaish, Ahmad Khalid, Ahmed Alsufyani, Ainhoa Valle Rubio, Akshay Bavikatte, Akshay Kumar, Al-Radjid Jamiri, Alberto de San Ildefonso Pereira, Alberto Porcu, Alberto Sartori, Aldo Rocca, Aleksandar Sretenovic, Alessandro Anselmo, Alessandro De Luca, Alexandros Charalabopoulos, Alexios Tzivanakis, Alfonso Bandin, Alfonso Nájar, Alice Frontali, Alsulaimani Faisal, Amaia Martínez Roldan, Amal Hamid, Ana André, Ana Minaya-Bravo, Andre Das, Andrea Bondurri, Andrea Costanzi, Andrea Lucchi, Andrei Mihailescu, Andrea Police, Andres Mendoza Zuchini, Angela Romano, Angelo Iossa, Antonella Chessa, Antonella Tromba, Antonio Castaldi, Antonio Brillantino, Antonio Ferronetti, Antonio Giuliani, Antonio Ramos-De la Medina, Antonio Tarasconi, Arcangelo Picciariello, Argyrios Ioannidis, Ari Leppäniemi, Arshad Rashid, Ashrarur Rahman Mitul, Asif Mehraj, Asim laharwal, Atif Iqbal, Athanasios Liarakos, Athanasios Marinis, Beatriz de Andrés-Asenjo, Belén Matías-García, Belinda De Simone, Ben Creavin, Ben Stubbs, Brian Goh, Branislav Jovanovic, Bruno Sensi, Carlo Gazia, Carlos Cerdán, Carlos Javier Gómez Díaz, Carlos Petrola Chacón, Carlos Yánez, Carmelo Lo Faro, Caroline Reinke, Casandra Dominguez, Charudutt Paranjape, Charlotte Thomas, Chia Chi Fung, Chiara De Lucia, Chiu Hiu Fung Jennifer, Christian Ovalle-Chao, Claudio Guerci, Cleo Kenington, Corina Gica, Cristina Folliero, Cristopher Varela, Daniel Popowich, Daniele Delogu, Daniele Zigiotto, Danilo Vinci, Dario D’Antonio, David Alessio Merlini, David Merlini, David Moro-Valdezate, Deborah Keller, Diana Cristiana Nicolaescu, Diego Sasia, Edgar Rodas, Dimitrios Linardoutsos, Domenico Russello, Pedro Alfonso Nájar-Castañeda, Habil Gregor Stavrou, Edoardo Rosso, Edoardo Saladino, Edoardo Ricciardi, Eduardo Smith-Singares, Efstratia Baili, Eleftheria Douka, Eleonora Guaitoli, Elisa Francone, Elisa Maria Vaterlini, Elisa Sefora Pierobon, Emilio Morales, Emilio Peña Ros, Enrico Benzoni, Enrico Erdas, Enrico Pinotti, Enrique Colás-Ruiz, Ernesto Laterza, Esteban Foianini, Eugenia Cardamone, Eugenio Licardie, Fabio Marino, Fahad Alsofyani, Fahad Qahtani, Farhan Khan, Fatlum Maraska, Fatmir Saliu, Fausto Madrid, Fausto Rosa, Federico Luvisetto, Felipe Alconchel, Felipe Pareja-Ciuro, Fernanda Neves, Ferdinando Agresta, Fernando Cordera, Fernando Pardo, Fernando Mendoza-Moreno, Fernando Munoz-Flores, Francesca Maria Silvestri, Francesca Paola Tropeano, Francesca Pecchini, Francesca Serio, Francesco Colombo, Francesco Di Marzo, Francesco Ferrara, Francesco Lancellotti, Francesco Litta, Francesco Martini, Francesco Roscio, Francisco Blanco-Antona, Francisco Javier Quezada Barcenas, Francisco Schlottmann, Gabriel Herrera-Almario, Gabrielle van Ramshorst, Gaetano Gallo, Gaetano Luglio, Georgios Kampouroglou, Georgios Papadopoulos, Gerardo Arredondo, Giacomo Calini, Giampaolo Formisano, Giampaolo Galiffa, Gian Marco Palini, Gianluca Colucci, Gianluca Pagano, Gianluca Vanni, Gianmaria Casoni Pattacini, Gianpiero Gravante, Giorgio Lisi, Giovanni Bellanova, Giovanni De Nobili, Giovanni Sammy Necchi, Giovanni Sinibaldi, Giulia Bacchiocchi, Giulia Bagaglini, Giulia Maggi, Giuliano Izzo, Giulio Argenio, Giuseppe Brisinda, Giuseppe Esposito, Giuseppe Frazzetta, Giuseppe Massimiliano De Luca, Giuseppe Nigri, Giuseppe Sica, Gonzalo Martin-Martin, Gustavo Armand Ugon, Gustavo Martinez-Mier, Gustavo Miguel Machain Vega, Gustavo Nari, Herald Nikaj, Ignacio Neri, Igor Alberdi San Roman, Iliya Fidoshev, Iñaki Martínez, Ionut Negoi, Irene Ortega, Irune Vicente Rodríguez, Isabel Cornejo, Ismael Mora-Guzmán, Issam al-Najami, Ivan Romic, Izaskun Balciscueta, James Olivier, Jan Lammel-Lindemann, Jana Dziakova, Javier Salinas, Jelena Pejanovic Jovanovic, Jeryl Anne Silvia Reyes, Joanne Salas, Jose Antonio Diaz-Elizondo, Jose Gustavo Parreira, Juan Bellido, Juan Salamea, Juan Carlos Martín del Olmo, Juliana María Ordoñez, Sofi Junaid, Justin Davies, Kapil Sahnan, Kebebe Bekele, Kelvin Voon, Leandro Siragusa, Lorenzo Petagna, Luca Ferrario, Luca Giordano, Luca Nespoli, Luca Pio, Lucia Moletta, Luciano Curella, Lucio Taglietti, Luigi Bonavina, Luigi Conti, Luis Eduardo Pérez-Sánchez, Luis Felipe Cabrera Vargas, Luis Sánchez-Guillén, Luis Tallon-Aguilar, Mansoor Khan, Marcello Giuseppe Spampinato, Marcelo Viola, Marcelo Viola Malet, Marco Angrisani, Marco Calussi, Marco Catarci, Marco Giordano, Marco Materazzo, Marco Milone, Marco Pellicciaro, Marco Vito Marino, María Daniela Moreno Villamizar, Maria Giulia Lolli, Maria Irene Bellini, Maria Lemma, Maria Michela Chiarello, Mario Montes-Manrique, Mario Rodriguez-Lopez, Mario Serradilla-Martín, Mark Peter, Marta Paniagua-García-Señoráns, Martin Rutegård, Martin Salö, Massimiliano Silveri, Massimiliano Veroux, Matteo Nardi, Matteo Rottoli, Matti Tolonen, Mauricio Pedraza Ciro, Mauricio Zuluaga, Maurizio Iacobone, Mauro Montuori, Mazin Ali, Melody García Domínguez, Menna Maria Paola, Micaela Piccoli, Michela Campanelli, Michele De Rosa, Michele Manigrasso, Michele Maruccia, Michele Torre, Michele Zuolo, Miguel Pera, Mihiri Weerasekera, Mikel Prieto, Min Myat Thway, Mohamed Shaat, Mohammad Azfar, Mostafa Shalaby, Muhammad Asif Raza, Muhammad Umar Younis, Muhammed Elhadi, Mujahid Ali, Musab AlThomali, Nadiah Al Amri, Nagendra Dudi-Venkata, Nahar Alselaim, Neil Smart, Nelson Trelles, Nicolò Falco, Niccolo Petrucciani, Nicola Antonacci, Nicola Cillara, Nicolae Gica, Nicolò Pecorelli, Nicolò Tamini, Nikolaos Machairas, Nura Feituri, Nuria Ortega Torrecilla, Octavio Avila Mercado, Ohood AlAamer, Oktay Irkorucu, Omar Alsherif, Oreste Claudio Buonomo, Orestes Valles-Guerra, Orestis Ioannidis, Oscar Isaac Hernández Palmas, Oscar Sanz Guadarrama, Osman Bozbiyik, Pablo Rodrigues, Pamela Milito, Paolo Panaccio, Panagiotis Dorovinis, Paola Prieto, Paolo Baroffio, Patrizia Marsanic, Pawel Ajawin, Peng Soon Koh, Pietro Anoldo, Piotr Major, Qasem Alharthi, Rashid Lui, Riccardo Caruso, Richard Brady, Rishi Rattan, Rishi Singhal, Roberta Angelico, Roberta Maria Isernia, Roberta Tutino, Roberto Peltrini, Rodrigo Tejos, Roosevelt Fajardo, Rossella Elia, Salvador Morales-Conde, Sami Benli, Sara Fuentes, Sara Gortázar de las Casas, Sara Ortiz de Guzmán Aragón, Sara Vertaldi, Selmy Awad, Sergio Gentilli, Sergio Alberto Weckmann Lujan, Serkan Tayar, Shabab Althobaiti, Silvia Di Giovanni, Soliman Ghedan, Sonia Pérez-Bertólez, Sonja Chiappetta, Spiros Delis, Stefano Scaringi, Süleyman Çetinkünar, Stylianos Kykalos, Syed Muhammad Ali, Sylvia Krivan, Tak Lit Derek Fung, Tarik Delko, Tatiana Nicolás López, Tercio De Campos, Teresa Calderón Duque, Teresa Perra, Theodore Liakakos, Theodoros Daskalakis, Tijmen Koëter, Tiku Zalla, Tomás Elosua González, Tommaso Campagnaro, Toure Alpha Oumar, Ugo Grossi, Valentina Sosa, Valentina Testa, Valentina Tomajer, Valeria Andriola, Valeria Tonini, Valerio Celentano, Valerio Voglino, Venkateswara Rao Katta, Víctor Hugo García Orozco, Victor Turrado-Rodriguez, Victor Visag-Castillo, Victoria Graham, Viktor Rachkov, Vincenzo Papagni, Vincenzo Vigorita, Virginia Jiménez Carneros, Vittoria Bellato, Wolf Bechstein, Yuksel Altinel, Zutoia Balciscueta

**Affiliations:** 1 General Surgery Unit, UOC di Chirurgia, Nicola Giannettasio Hospital, Via Ippocrate, 87064 Corigliano-Rossano, CS Italy; 2 La Sapienza University, Rome, Italy; 3 Division of Hepatobiliary and Liver Transplantation Surgery, A.O.R.N. Cardarelli, Naples, Italy; 4 Department of Surgical Science, University of Cagliari, Cagliari, Italy; 5 Department of Surgery, Madonna del Soccorso General Hospital, San Benedetto del Tronto, Italy; 6 Hepatobiliary division, Hospital del Mar, Universitat Pompeu Fabra, Barcelona, Spain; 7 Department of Advanced Medical and Surgical Sciences, Universitá degli Studi della Campania “Luigi Vanvitelli”, Policlinico CS, Piazza Miraglia 2, 80138 Naples, Italy; 8 Colorectal Surgery, Vall d’Hebron University Hospital, Barcelona, Spain

## Abstract

**Background:**

In 2020, ACIE Appy study showed that COVID-19 pandemic heavily affected the management of patients with acute appendicitis (AA) worldwide, with an increased rate of non-operative management (NOM) strategies and a trend toward open surgery due to concern of virus transmission by laparoscopy and controversial recommendations on this issue. The aim of this study was to survey again the same group of surgeons to assess if any difference in management attitudes of AA had occurred in the later stages of the outbreak.

**Methods:**

From August 15 to September 30, 2021, an online questionnaire was sent to all 709 participants of the ACIE Appy study. The questionnaire included questions on personal protective equipment (PPE), local policies and screening for SARS-CoV-2 infection, NOM, surgical approach and disease presentations in 2021. The results were compared with the results from the previous study.

**Results:**

A total of 476 answers were collected (response rate 67.1%). Screening policies were significatively improved with most patients screened regardless of symptoms (89.5% vs. 37.4%) with PCR and antigenic test as the preferred test (74.1% vs. 26.3%). More patients tested positive before surgery and commercial systems were the preferred ones to filter smoke plumes during laparoscopy. Laparoscopic appendicectomy was the first option in the treatment of AA, with a declined use of NOM.

**Conclusion:**

Management of AA has improved in the last waves of pandemic. Increased evidence regarding SARS-COV-2 infection along with a timely healthcare systems response has been translated into tailored attitudes and a better care for patients with AA worldwide.

## Introduction

COVID-19 pandemic has heavily impacted on surgical services and surgery [1, 2]. Since the declaration of pandemic by WHO on the March 12, 2020, 435,626,514 confirmed cases and 5,952,215 deaths have been reported globally [3]. Healthcare systems shifted resources and personnel to manage the increasing number of COVID-19 patients, cancelling or postponing elective operations and outpatient clinics, reducing surgical beds as a tailored strategy to avoid unnecessary resource consumption and mitigate the risk of SARS-COV-2 infection in surgical patients [4]. In urgent surgical diseases, such as acute cholecystitis, acute diverticulitis and acute appendicitis (AA), national and international surgical societies recommended to improve non-operative management (NOM), whenever applicable, avoiding admission in the hospital and supporting alternative strategies such as phone and remote-follow-up [5–8].

In 2020, the “Association of Italian Surgeons in Europe” (*Associazione Chirurghi Italiani in Europa*, ACIE) explored the global attitudes in the management of AA on an international cohort of 709 surgeons in the Appy study [9], showing a statistically significant shift toward NOM during the first phase of the outbreak in comparison with the pre-pandemic period (23.7 and 5.3 percent vs. 6.6 and 2.4 percent, respectively, both *P* < 0.001) with one-third of respondents moving toward open surgery in line with the initial recommendations/guidelines released in the early stages of the pandemic.

With the evolution of pandemic and a major knowledge of the disease, several strategies are now in place to mitigate the risk and might have produced an important effect in the management of AA, still the most common surgical abdominal emergency with a long-life risk of 8–9% [10].

In the present study (ACIE Appy II), we surveyed the same sample of surgeons to explore if any differences occurred in the management of AA in the last waves of pandemics during 2021.

## Method

A follow-up internet-based survey based on a previous research project [9] from the ACIE study group was carried out to investigate the impact of the COVID-19 pandemic over the clinical decision for patients with AA, one year after the beginning of the pandemic. An online questionnaire was sent to all 709 participants of the ACIE Appy study by email (Appendix 2). The data sampling collected information from Surgical trainees or certified Surgeons across Europe, Asia, Africa, Oceania, North and South America. The purpose of the study was communicated beforehand to each participant, whose enrollment was voluntary as no incentives were offered to collaborate with the study.

### Questionnaire development and composition

Based on the previously used strategy [9], the components and topics for the questionnaire were developed by the steering committee using web-based and remote discussion. The technical functionality of the electronic questionnaire was tested before sending the invitations. Names, locations, and baseline information were stored with the questionnaire. Once an agreement was reached, the questionnaire was completed using Google Form [*The COVID-19 Appy-2 Study Form*] survey software (Google LLC, Mountain View, California US).

The questionnaire included 5 Sections, with 36 closed-ended questions in total. The first three sections included general questions about the hospital organization and screening policies; personal protective equipment and personal attitudes about the management of AA. The fourth and fifth ones focused on the real-life analysis of patient presentation and management strategies of patients with AA one year after the beginning of the COVID-19 pandemic.

Uncomplicated appendicitis was defined as appendicitis without abscess, whereas complicated appendicitis included the presence of an intraabdominal abscess or free perforation with diffuse peritonitis. Non-operative management (NOM) was defined as conservative management with antibiotics; this could include percutaneous abscess drainage.

The list of alternatives for every single quantitative question included a percentage category as follows: “≤ 25%”, “26–50%”, “51–75%”, “76–100%”. The steering committee decided to use ranges of predetermined percentages in order to allow an easier aggregation and analysis of the information collected.

The estimated time to complete the survey was 8–10 min. The aim was to define the status of the management of AA one year after the beginning of the pandemic as compared with the pandemic period.

### Study circulation

From August 15, 2021, the questionnaire was online and open to completion until September 30, 2021. The link was sent to all 709 ACIE Appy participants by email. Two other remainders were sent, always by email, to maximize the response rate.

### Data handling and extraction

A member of the steering committee (MP) downloaded the questions and shared them with the other members for data analysis and discussion. Multiple entries from the same individual or members of the same surgical unit were manually searched and eliminated if contradictory findings were observed.

### Statistical analysis

Descriptive data are presented as numbers and proportions for categorical variables. Contingency tables and the Chi-square test were used for the comparisons. Statistical analyses were performed in Stata version 16.0 (StataCorp), and nominal two-sided *P* < 0.05 values were considered statistically significant.

## Results

### Baseline information

Overall, in 2021, 476 answers (response rate 67.1%) were received from 59 Countries. Most respondents were from countries that were the most affected at the time of the first wave of the pandemic and collaborate with the previous survey. A total of 189 answers (39.7%) were returned from Italy and 64 (13.4%) from Spain, summing together about half of the answers received.

#### Hospital organization and screening policies

**Fig. 1:**
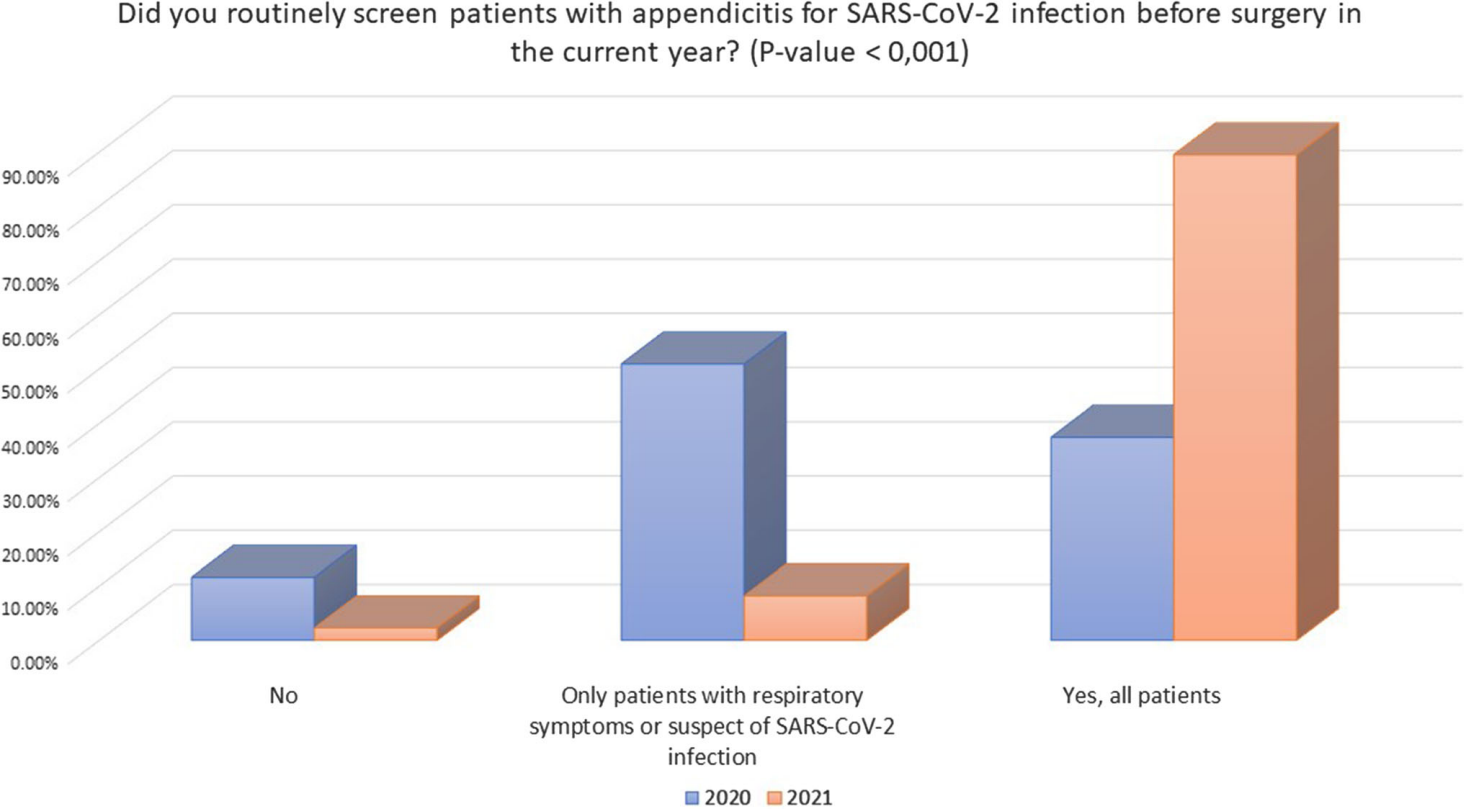
Screening policies in patients with acute appendicitis for SARS-CoV-2 infection during 2020 and 2021

**Fig. 2:**
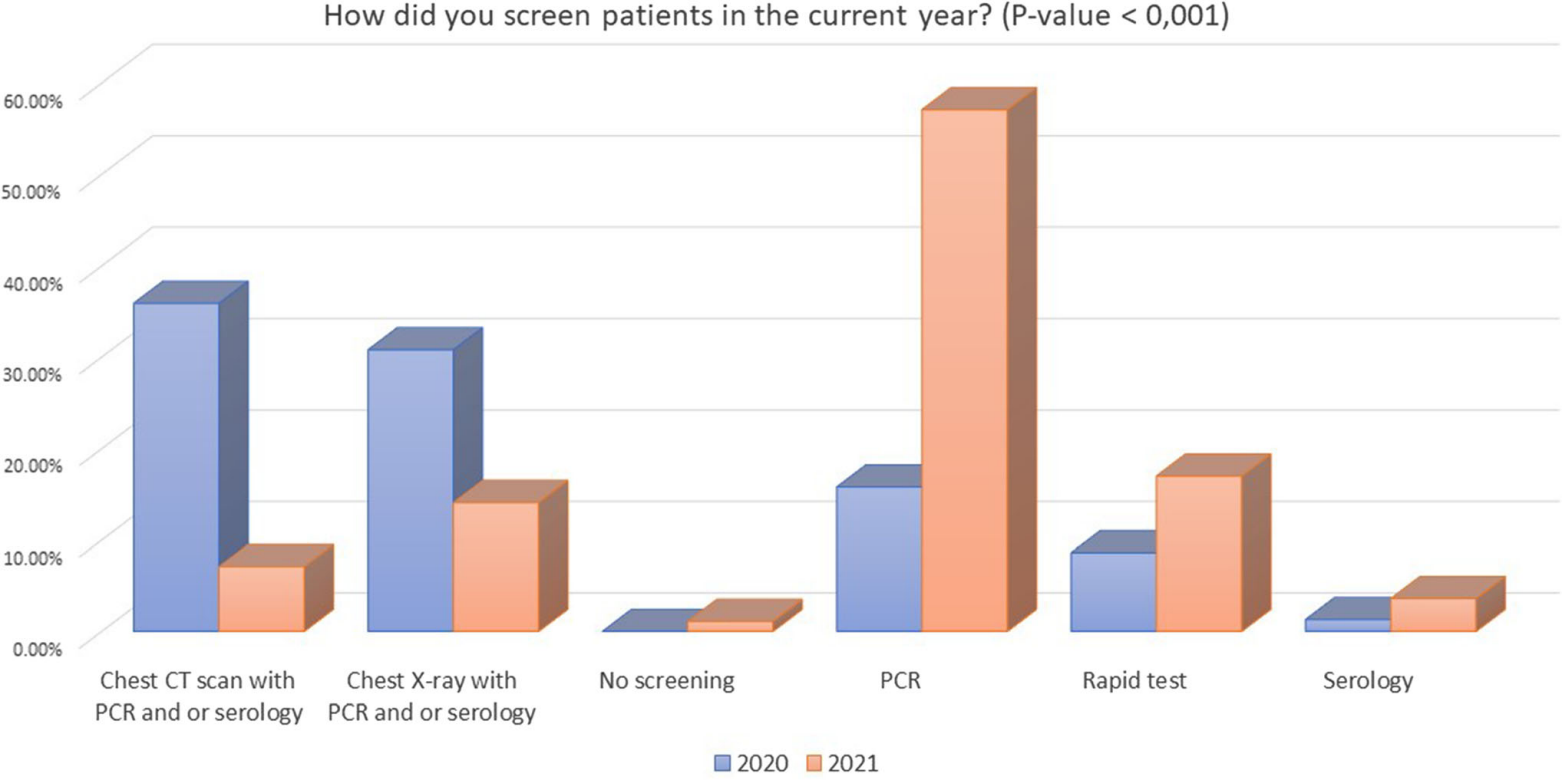
Types of screening in patients with acute appendicitis for SARS-CoV-2 infection during 2020 and 2021

Baseline characteristics of included hospitals in 2021 were comparable to those included in the previous survey from 2020 with 91.8% hospitals attending COVID-19 patients and 8.1% not specifically treating COVID-19 patients (Table 1). The screening policy in 2021 showed significant differences in comparison with 2020 with a higher percentage of systematic SARS-CoV-2 screening for all patients with AA (89.5% vs. 37.4%, *P* < 0.001) (Fig. 1) and not only to symptomatic subjects. Moreover, the number of patients screened exclusively with PCR and antigenic tests in 2021 was significantly higher in comparison with 2020 (74.1% vs. 26.3%, *P* < 0.001) when 66.7% of patients were screened with a chest X-ray or computed tomography (CT) in addition to PCR or serological tests (Fig. 2).

**Table 1:** Changes in the screening policies and use of personal protective equipment (PPE) from pandemic 2020 to 2021

Query	Options	Study period: pandemic 2020 *N* = 701	Study period: 2021 *N* = 476	*P* value
Hospital changes in relation to COVID-19 pandemic	My hospital does not treat COVID-19 patients	8.1% (57)	8.0% (38)	0.27
	My hospital has restricted areas dedicated to COVID-19 patients	83.0% (582)	85.7% (408)	
	My hospital is exclusively dedicated to COVID-19 patients	8.8% (62)	6.3% (30)	
Screening for SARS-CoV-2 infection in patients with appendicitis during the pandemic	No	11.6% (81)	2.3% (11)	**< 0.001**
	Only patients with respiratory symptoms or suspect of SARS-CoV-2 infection	50.9% (357)	8.2% (39)	
	Yes, all patients	37.4% (262)	89.5% (426)	
Methods of screening	Chest CT scan with PCR and or serology	38.8% (252)	7.1% (34)	**< 0.001**
	Chest X-ray with PCR and or serology	33.3% (216)	14.1% (67)	
	No screening	0.0% (0)	1.1% (5)	
	PCR or antigenic test	26.3% (171)	74.1% (353)	
	Serology	1.3% (9)	3.6% (17)	

Bold numbers refers to significant values (when p<0.05)

#### Personal protective equipment (PPE)

The survey on changes in the use of PPE showed that in 2021, a higher number of surgeons started to use no specific personal protective devices (12.2% vs. 0% *P* < 0.001), even in untested patients (2.9% vs. 0%, *P* < 0.001) (Table 2) in comparison with 2020. Conversely, in COVID-19 positive patients, specific personal protections were always used, with an increase in the utilization of FFP2/FFP3 mask in 2021 (61.2% vs. 59.8, *P* < 0.001).

**Table 2:** Changes in the use of personal protective equipment (PPE) during COVID-19 pandemic, according to patient SARS-CoV2 status, from pandemic 2020 to 2021

Query	Options	Study period: pandemic 2020	Study period: 2021	*P* value
Personal protective devices used in SARS-CoV-2 negative patients	Face mask (FFP2/FFP3)	54.9% (239)	63.3% (302)	< 0.001
	N95 face mask	39.5% (172)	23.9% (114)	
	No personal protective devices	0.0% (0)	12.2% (58)	
Personal protective devices used in SARS-CoV-2 untested patients	Face mask (FFP2/FFP3)	62.0% (354)	57.4% (273)	< 0.001
	N95 face mask	28.5% (200)	26.5% (126)	
	No personal protective devices	0.0% (0)	2.9% (14)	
	We accept negative tested patients only	0.0% (0)	13.2% (63)	
Personal protective devices used in SARS-CoV-2 positive patients	Face mask (FFP2/FFP3)	59.8% (419)	61.2% (291)	< 0.001
	N95 mask and goggles	34.7% (243)	31.1% (148)	
	No personal protective devices	0.0% (0)	0.4% (2)	
	We accepted negative tested patients only	0.0% (0)	7.1% (34)	

#### Personal attitude: operative versus non-operative management of acute appendicitis

**Fig. 3:**
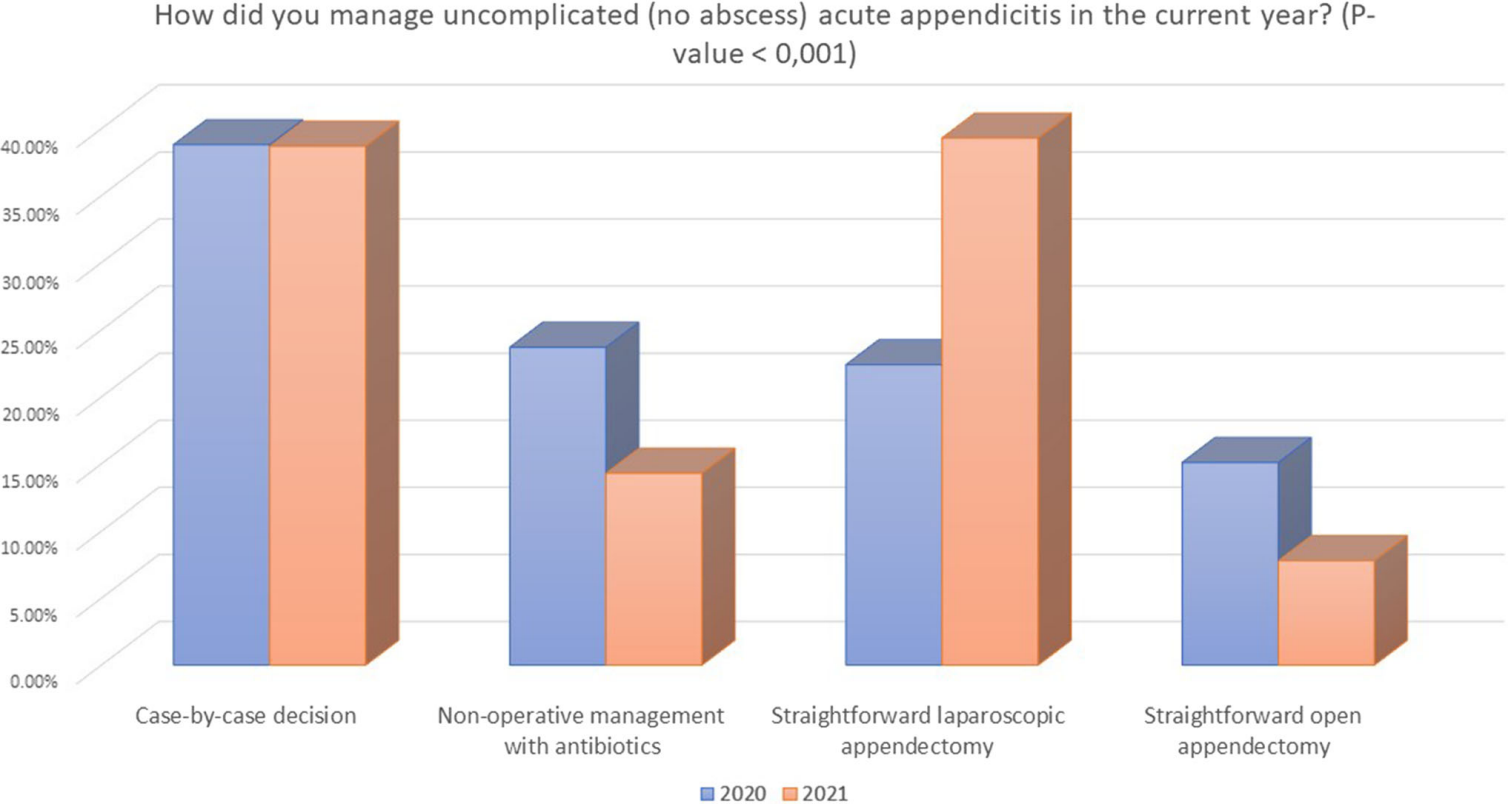
Management of uncomplicated appendicitis in 2020 and 2021

In patients with uncomplicated appendicitis (no right iliac fossa abscess), in 2021, a higher percentage of surgeons did not change their attitude in the management of these patients (55.7% vs. 42.5%, *P* < 0.001) (Table 3), whereas 13.9% still admitted changing their treatment strategy, especially in COVID-19 positive or untested patients (30.5%) (Fig. 3). The rate of subjects treated with NOM significantly decreased (14.3% vs. 23.7%, *P* < 0.001), whereas there was a significant raise in the number of surgeons performing straightforward laparoscopic appendectomy (39.3% vs. 22.4%, *P* < 0.001).

**Table 3:** Changes in personal attitude for acute appendicitis from pandemic 2020 to 2021

Query	Options	Study period: pandemic 2020 *N* = 701	Study period: 2021 *N* = 476	*P* value
Management of uncomplicated acute appendicitis during the pandemic: did you change your attitude?	No	42.5% (298)	55.7% (265)	**< 0.001**
	Yes	28.4% (199)	13.9% (66)	
	Yes, only in COVID-19 + patients	29.0% (203)	30.5% (145)	
Management of uncomplicated acute appendicitis (no abscess)	Case-by-case decision	38.8% (272)	38.7% (184)	**< 0.001**
	Non-operative management with antibiotics	23.7% (166)	14.3% (68)	
	Straightforward laparoscopic appendectomy	22.4% (157)	39.3% (187)	
	Straightforward open appendectomy	15.1% (106)	7.8% (37)	
Management of complicated acute appendicitis (abscess) during the pandemic: did you change your attitude?	No	47.4% (332)	67.9% (323)	**< 0.001**
	Yes	28.1% (197)	8.8% (42)	
	Yes, only in COVID-19 + patients	24.4% (171)	23.3% (111)	
Management of complicated acute appendicitis (abscess)	Non-operative management with antibiotics ± percutaneous drainage	38.1% (266)	22.8% (109)	**< 0.001**
	Straightforward laparoscopic appendectomy	33.5% (235)	64.9% (309)	
	Straightforward open appendectomy	28.0% (196)	12.2% (58)	

Bold numbers refers to significant values (when p<0.05)

In case of appendicitis complicated by right iliac fossa abscess, once again, in 2021, a higher percentage of surgeons did not change their attitude in the management of these patients (67.9% vs. 47.4%, *P* < 0.001). Additionally, similarly to uncomplicated appendicitis, the rate of subjects treated with NOM significantly decreased (20% vs. 32.7%, *P* < 0.001), while surgeons tended to perform more straightforward laparoscopic appendectomy (64.9% vs. 33.5%, *P* < 0.001).

#### Personal attitude: surgical approach

In 2021, surgeons tended to change less frequently their standard surgical approach (86.8% vs. 61.1%, *P* < 0.001) from laparoscopic to open or vice versa than in 2020 (Table 4).

**Table 4:** Changes in surgical approach for acute appendicitis and aspiration of plumes from pandemic 2020 to 2021

Query	Options	Study period: pandemic 2020 *N* = 701	Study period: 2021 *N* = 476	*P* value
Did you change the surgical approach (open vs. laparoscopic) to appendectomy due to the COVID-19 pandemic?	No	61.1% (428)	86.8% (413)	**< 0.001**
	Yes, from laparoscopic to open	36.4% (255)	12.0% (57)	
	Yes, from open to laparoscopic	2.3% (16)	1.3% (6)	
How did you operate on COVID-19 positive patients with appendicitis?	Always open surgery, personal preference	29.5% (207)	13.9% (66)	**< 0.001**
	I did not operate on COVID-19 positive patients in the current year	0.0% (0)	16.8% (80)	
	I would use laparoscopic, but I do not have devices for pneumoperitoneum/smoke evacuation	20.4% (143)	10.3% (49)	
	Laparoscopic surgery	48.6% (341)	59.0% (281)	
How did you operate on COVID-19 untested patients with appendicitis?	Always open surgery, I prefer	27.7% (194)	10.5% (50)	**< 0.001**
	I did not operate on COVID-19 untested patients in the current year	0.0% (0)	32.8% (156)	
	I would use laparoscopic, but I do not have devices for pneumoperitoneum/smoke evacuation	17.0% (119)	6.9% (33)	
	Laparoscopic surgery	49.2% (345)	49.8% (233)	
If laparoscopic appendectomy was performed, did you use any filter system?	No	25.8% (181)	31.7% (151)	**< 0.001**
	Yes	73.8% (510)	68.2% (325)	
If any evacuation system, which type of device did you employ?	Commercially available	56.3% (395)	64.6% (306)	**< 0.001**
	Homemade	33.9% (237)	21.9% (104)	

Bold numbers refers to significant values (when p<0.05)

There was an increase in surgeons that did not have to operate con COVID-19 positive patients (16.8% vs. 0%, *P* < 0.0001), whereas the number of those performing laparoscopic appendectomy increased (59% vs. 48.6%, *P* < 0.001).

If a laparoscopic appendectomy was performed, a higher number of survey respondents in 2021 stated not to use specific smoke filter system (31.7% vs. 25.8%, *P* < 0.001). However, when such kind of devices were used, they were more frequently represented by commercially available systems (64.6% vs. 56.3%, *P* < 0.001) than homemade package.

#### Changes in patient presentation from 2020 to 2021 at participants Institutions

In 2021, there was a higher percentage of patients with AA that tested positive for SARS-CoV-2 before or after surgery (*P* < 0.001) (Table 5).

**Table 5:** Changes in epidemiology from 2020 to 2021

Query	Options	Study period: pandemic 2020	Study period: 2021	*P* value
Did any patient referred for acute appendicitis test positive for SARS-CoV-2 before surgery?	0	67.5% (473)	18.3% (87)	**< 0.001**
	1–5%	27.1% (190)	63.4% (302)	
	6–10%	3.3% (23)	13.7% (65)	
	> 10%	1.0% (7)	4.6% (22)	
Did any COVID-19 negative patient referred for acute appendicitis later test positive for SARS-CoV-2?	0	70.3% (493)	39.1% (186)	**< 0.001**
	1–5%	20.7% (145)	47.1% (224)	
	6–10%	2.9% (20)	7.8% (37)	
	> 10%	4.0% (28)	6.1% (29)	
How many patients with acute appendicitis have been referred to your hospital during the last month?	< 5	38.7% (271)	16.0% (76)	**< 0.001**
	5–10	33.4% (234)	41.2% (196)	
	11–20	16.5% (116)	42.9% (204)	
	> 20	10.4% (73)	0.0% (0)	
How many patients with uncomplicated acute appendicitis (no abscess) are treated with a non-operative management with antibiotics?	≤ 25%	59.5% (417)	73.3% (349)	**< 0.001**
	26–50%	15.7% (110)	16.2% (77)	
	51–75%	11.6% (81)	6.9% (33)	
	76–100%	12.0% (84)	3.6% (17)	
How many patients with uncomplicated acute appendicitis (no abscess) treated conservatively with antibiotics are currently sent home and followed up at the outpatient clinic?	≤ 25%	66.3% (465)	72.1% (343)	**0.007**
	26–50%	9.5% (67)	6.5% (31)	
	51–75%	9.7% (68)	9% (43)	
	76–100%	2% (14)	0.0% (0)	
How many patients with complicated acute appendicitis (with abscess) currently, undergo conservative treatment with antibiotics ± percutaneous drainage?	≤ 25%	66.8% (468)	78.2% (372)	**0.002**
	26–50%	12.1% (85)	9.9% (47)	
	51–75%	9.1% (64)	6.7% (32)	
	76–100%	9.7% (68)	5.3% (25)	
How many patients with acute appendicitis treated with surgery currently undergo open appendectomy?	≤ 25%	53.2% (373)	72.7% (346)	**< 0.001**
	26–50%	13.1% (92)	9.7% (46)	
	51–75%	9.7% (68)	6.7% (32)	
	76–100%	22.5% (158)	10.9% (52)	

Bold numbers refers to significant values (when p<0.05)

**Fig. 4:**
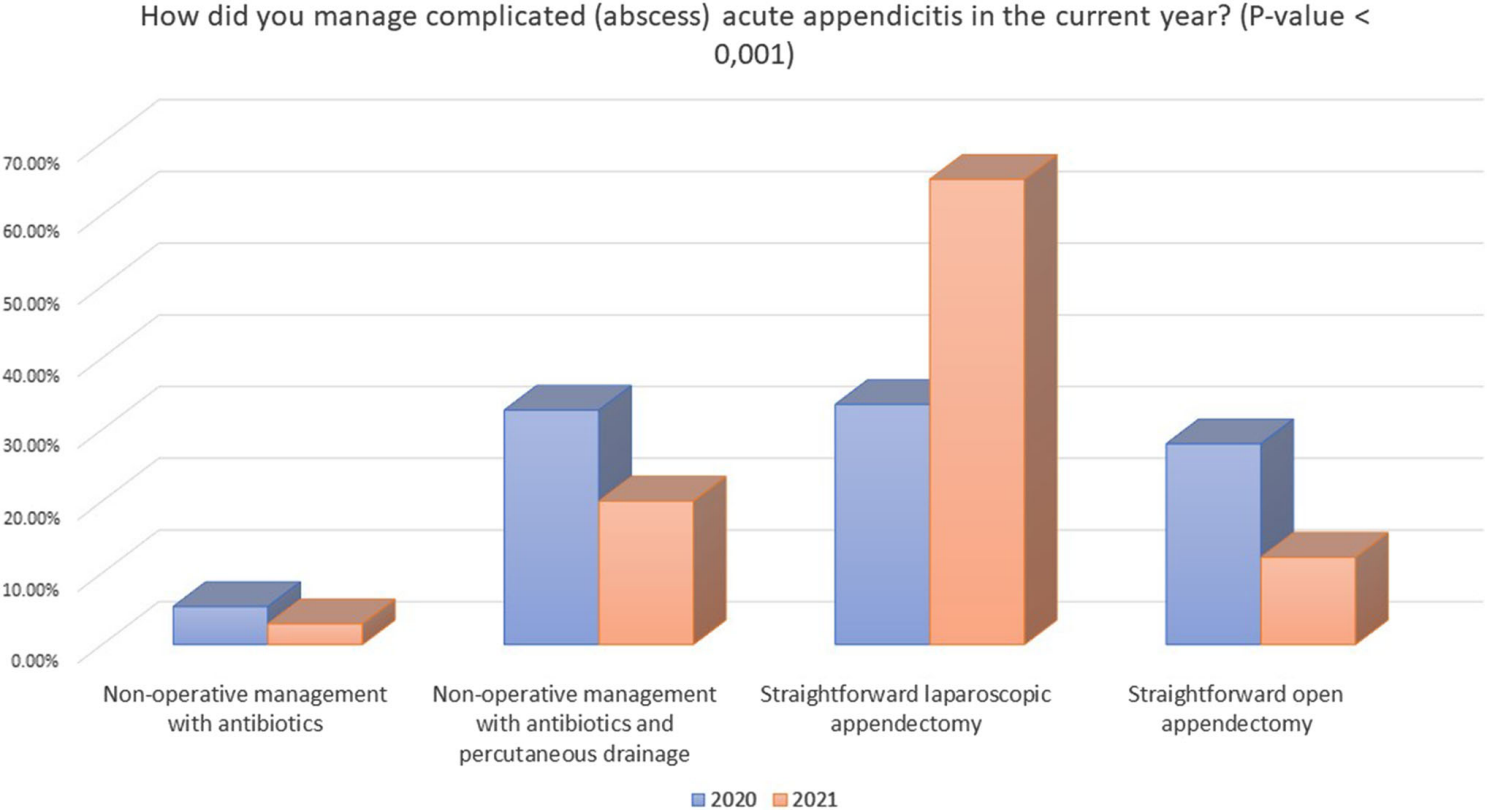
Management of complicated appendicitis in 2020 and 2021

Respondents from 2021 tended to use less frequently a NOM and send patients home in uncomplicated appendicitis than those from 2020 (*P* < 0.001 and *P* = 0.007, respectively). Conversely, more patients with complicated appendicitis were treated with conservative treatment with antibiotics ± percutaneous drainage (*P* = 0.002) (Fig. 4).

## Discussion

Our study revealed some attitudinal changes in the management of AA in 2021 in comparison with the first waves of pandemic in 2020. Improvement of the knowledge of SARS-CoV-2 infection, a timely healthcare systems’ response in term of screening and a wider availability of personal protective equipment (PPE) are the probable causes. The knowledge of the evolution trends in AA management may helpful for countries interested by a SARS-CoV-2 flare-up and may support healthcare stakeholders to inform pandemic plans for future outbreaks.

### Local policies and screening

Screening policies were significatively improved with most patients screened regardless of symptoms (89.5% vs. 37.4%) with PCR and antigenic tests as the preferred test (74.1% vs. 26.3%). More patients tested positive before surgery, but this could reflect a systematic screening of patients admitted in the hospital, not possible for reduced availability of diagnostic tests in the first months of the pandemic, where half of the responders screened only patients suspected or with respiratory symptoms. Because COVID-19 patients undergoing surgery have an estimated mortality of 23.8% and a 51.2% of pulmonary complications [11], a patient with AA tested positive for SARS-CoV-2 may benefit from a NOM trial to avoid surgery.

### Open versus laparoscopic appendicectomy

During the first wave of the pandemic, in the perspective of a short-term outbreak and due to the concern potential spreading of SARS-COV-2 particles by smoke plumes and pneumoperitoneum during laparoscopy, open surgery was initially suggested by some surgical societies and some authors [12–15]. The effect was evident in our previous study, with one third of responders moving toward an open approach. Although SARS-COV-2 RNA has been detected in the peritoneal fluid [16, 17] and transmission is biologically plausible [18], no case of transmission by laparoscopy has been reported. Moreover, smoke plumes evacuation might be more challenging in open surgery and several homemade and commercial ultrafiltration systems are now available to err on the side of safety [19–22], although some of these systems may be time and cost-consuming, especially in emergency surgery by an already stretched staff [23].

While 68.2% of responders use ultrafiltration system during laparoscopic appendicectomy systematically or in COVID-19 patients, one third of responders (31.7%) declare not to use ultrafiltration device during laparoscopy, and this percentage was higher than in previous study (31.7% vs. 25.8%). This may reveal a reduced perceived risk of transmission associated to minimally invasive surgery or a low estimate of these devices as crucial mitigation strategy in operative room.

Laparoscopic appendicectomy is now unanimously recommended for its advantages, including shorter recovery time, better diagnostic accuracy and the possibility to be performed as ambulatory appendectomy [24–26] to reduce the risk of system overburden. In the light of the long-term pandemic, a 30% of recurrence in the NOM patients as reported by the CODA trial [27] may represents a further point in favor of laparoscopy, reducing costs and risk of SARS-CoV-2 exposure in patients attending emergency department [28]. In comparison with the previous survey, the proportion of centers performing 76–100% of appendicectomy by open approach declined significantly moving from 22.5 to 10.9%, a percentage similar to the declared 9.1% of pre-pandemic times [9].

### Non-operative management (NOM)

The pandemic scenario has had a major impact on presentation rate and strategies of AA worldwide. A meta-analysis on this topic [29] showed a significatively increased use of conservative management of AA in all ages during the outbreak than before. Especially in the more troublesome times of pandemic, NOM remained a reasonable option in patients with AA, with fewer complications and shorter length of stay [30, 31] although with a low effective rate than surgery [32]. In a recent meta-analysis on the role of NOM in the COVID-19 era including 2140 patients, 44.8% of patients had a trial of NOM, with a failure rate of 16.4% and a complication rate of 4.5% with no mortality [33].

Interestingly, only 10.5% of respondents used NOM in more than half (51–100%) of cases with uncomplicated AA, a twofold reduction in comparison with the 23.6% of the previous study. This change may reflect a shift toward the use of laparoscopic appendicectomy as the first option, due to its superiority in the definitive control of the disease [34, 35], a better organization of the healthcare systems and less concern about the risk of viral spreading by minimally invasive surgery.

### Appendicular abscess

A higher rate of complicated appendicitis during pandemic have been reported by several authors [36–38]. However, due to the decrease in the overall number of cases, this might be related to a prehospital selection bias: as many cases of uncomplicated AA probably settled outside the hospital by antibiotic or spontaneously [39] the rate of those decreased, while complicated AAs are admitted at the same number with an apparent increase in their rate.

NOM may be an option in case of appendicular abscess with a failure rate of 7.4% and the need for percutaneous drain in 19.8%. However, laparoscopy shows the best outcomes in terms of readmission and reintervention [40]. Only 22.8% of responders applied NOM ± percutaneous drainage strategy in appendicular abscess versus the 38.1% from the previous study. These may reflect again an implementation of surgical system response to pandemic, despite the increasing number of AA treated, as the 84.1% of centers declares to treat 5–20 cases per month versus 49.9% in 2020.

### Study limitations

This study presents some limitations. For a fair comparison, we used the same self-selected sample of the first study: the missed participation in the first survey by surgeons as their countries were marginally interested by the first COVID-19 waves did not consent their participation in the present study, introducing a selection bias. However, most of the respondents were from countries (Italy, Spain) heavily impacted by all pandemic waves, so that important data about the change in clinical decision-making in the management of acute appendicitis during the different phases of pandemic can be obtained. To keep pragmatic the design of the study, we did not investigate about SARS-CoV-2 vaccination among responders and patients treated, so that important information about the system response in 2021 was not captured. To our knowledge, this is the first survey re-evaluating for the second time the changes in the attitudes in the management of AA occurred in the COVID-19 pandemic worldwide.

## Conclusions

The management of acute appendicitis in the last part of pandemic has been improved moving toward pre-pandemic standard due to a better understanding of SARS-CoV-2 infection and improved response by healthcare systems worldwide.

## APPENDIX 1

### The ACIE appy-2 collaborative study group

Abba Julio, Abdullah Alshamrani, Abdullah Alturkistani, Abdulrahman Alghamdi, Abdulrahman Almalki, Adam Orengia, Adnan Kuvvetli, Adolfo Pisanu, Adrian Smith, Adriana Michelle Treviño Figueroa, Aeris Jane Nacion, Ahmad Alhazmi, Ahmad Bouhuwaish, Ahmad Khalid, Ahmed Alsufyani, Ainhoa Valle Rubio, Akshay Bavikatte, Akshay Kumar, Al-Radjid Jamiri, Alberto de San Ildefonso Pereira, Alberto Porcu, Alberto Sartori, Aldo Rocca, Aleksandar Sretenovic, Alessandro Anselmo, Alessandro De Luca, Alexandros Charalabopoulos, Alexios Tzivanakis, Alfonso Bandin, Alfonso Nájar, Alice Frontali, Alsulaimani Faisal, Amaia Martínez Roldan, Amal Hamid, Ana André, Ana Minaya-Bravo, Andre Das, Andrea Bondurri, Andrea Costanzi, Andrea Lucchi, Andrei Mihailescu, Andrea Police, Andres Mendoza Zuchini, Angela Romano, Angelo Iossa, Antonella Chessa, Antonella Tromba, Antonio Castaldi, Antonio Brillantino, Antonio Ferronetti, Antonio Giuliani, Antonio Ramos-De la Medina, Antonio Tarasconi, Arcangelo Picciariello, Argyrios Ioannidis, Ari Leppäniemi, Arshad Rashid, Ashrarur Rahman Mitul, Asif Mehraj, Asim laharwal, Atif Iqbal, Athanasios Liarakos, Athanasios Marinis, Beatriz de Andrés-Asenjo, Belén Matías-García, Belinda De Simone, Ben Creavin, Ben Stubbs, Brian Goh, Branislav Jovanovic, Bruno Sensi, Carlo Gazia, Carlos Cerdán, Carlos Javier Gómez Díaz, Carlos Petrola Chacón, Carlos Yánez, Carmelo Lo Faro, Caroline Reinke, Casandra Dominguez, Charudutt Paranjape, Charlotte Thomas, Chia Chi Fung, Chiara De Lucia, Chiu Hiu Fung Jennifer, Christian Ovalle-Chao, Claudio Guerci, Cleo Kenington, Corina Gica, Cristina Folliero, Cristopher Varela, Daniel Popowich, Daniele Delogu, Daniele Zigiotto, Danilo Vinci, Dario D’Antonio, David Alessio Merlini, David Merlini, David Moro-Valdezate, Deborah Keller, Diana Cristiana Nicolaescu, Diego Sasia, Edgar Rodas, Dimitrios Linardoutsos, Domenico Russello, Pedro Alfonso Nájar-Castañeda, Habil Gregor Stavrou, Edoardo Rosso, Edoardo Saladino, Edoardo Ricciardi, Eduardo Smith-Singares, Efstratia Baili, Eleftheria Douka, Eleonora Guaitoli, Elisa Francone, Elisa Maria Vaterlini, Elisa Sefora Pierobon, Emilio Morales, Emilio Peña Ros, Enrico Benzoni, Enrico Erdas, Enrico Pinotti, Enrique Colás-Ruiz, Ernesto Laterza, Esteban Foianini, Eugenia Cardamone, Eugenio Licardie, Fabio Marino, Fahad Alsofyani, Fahad Qahtani, Farhan Khan, Fatlum Maraska, Fatmir Saliu, Fausto Madrid, Fausto Rosa, Federico Luvisetto, Felipe Alconchel, Felipe Pareja-Ciuro, Fernanda Neves, Ferdinando Agresta, Fernando Cordera, Fernando Pardo, Fernando Mendoza-Moreno, Fernando Munoz-Flores, Francesca Maria Silvestri, Francesca Paola Tropeano, Francesca Pecchini, Francesca Serio, Francesco Colombo, Francesco Di Marzo, Francesco Ferrara, Francesco Lancellotti, Francesco Litta, Francesco Martini, Francesco Roscio, Francisco Blanco-Antona, Francisco Javier Quezada Barcenas, Francisco Schlottmann, Gabriel Herrera-Almario, Gabrielle van Ramshorst, Gaetano Gallo, Gaetano Luglio, Georgios Kampouroglou, Georgios Papadopoulos, Gerardo Arredondo, Giacomo Calini, Giampaolo Formisano, Giampaolo Galiffa, Gian Marco Palini, Gianluca Colucci, Gianluca Pagano, Gianluca Vanni, Gianmaria Casoni Pattacini, Gianpiero Gravante, Giorgio Lisi, Giovanni Bellanova, Giovanni De Nobili, Giovanni Sammy Necchi, Giovanni Sinibaldi, Giulia Bacchiocchi, Giulia Bagaglini, Giulia Maggi, Giuliano Izzo, Giulio Argenio, Giuseppe Brisinda, Giuseppe Esposito, Giuseppe Frazzetta, Giuseppe Massimiliano De Luca, Giuseppe Nigri, Giuseppe Sica, Gonzalo Martin-Martin, Gustavo Armand Ugon, Gustavo Martinez-Mier, Gustavo Miguel Machain Vega, Gustavo Nari, Herald Nikaj, Ignacio Neri, Igor Alberdi San Roman, Iliya Fidoshev, Iñaki Martínez, Ionut Negoi, Irene Ortega, Irune Vicente Rodríguez, Isabel Cornejo, Ismael Mora-Guzmán, Issam al-Najami, Ivan Romic, Izaskun Balciscueta, James Olivier, Jan Lammel-Lindemann, Jana Dziakova, Javier Salinas, Jelena Pejanovic Jovanovic, Jeryl Anne Silvia Reyes, Joanne Salas, Jose Antonio Diaz-Elizondo, Jose Gustavo Parreira, Juan Bellido, Juan Salamea, Juan Carlos Martín del Olmo, Juliana María Ordoñez, Sofi Junaid, Justin Davies, Kapil Sahnan, Kebebe Bekele, Kelvin Voon, Leandro Siragusa, Lorenzo Petagna, Luca Ferrario, Luca Giordano, Luca Nespoli, Luca Pio, Lucia Moletta, Luciano Curella, Lucio Taglietti, Luigi Bonavina, Luigi Conti, Luis Eduardo Pérez-Sánchez, Luis Felipe Cabrera Vargas, Luis Sánchez-Guillén, Luis Tallon-Aguilar, Mansoor Khan, Marcello Giuseppe Spampinato, Marcelo Viola, Marcelo Viola Malet, Marco Angrisani, Marco Calussi, Marco Catarci, Marco Giordano, Marco Materazzo, Marco Milone, Marco Pellicciaro, Marco Vito Marino, María Daniela Moreno Villamizar, Maria Giulia Lolli, Maria Irene Bellini, Maria Lemma, Maria Michela Chiarello, Mario Montes-Manrique, Mario Rodriguez-Lopez, Mario Serradilla-Martín, Mark Peter, Marta Paniagua-García-Señoráns, Martin Rutegård, Martin Salö, Massimiliano Silveri, Massimiliano Veroux, Matteo Nardi, Matteo Rottoli, Matti Tolonen, Mauricio Pedraza Ciro, Mauricio Zuluaga, Maurizio Iacobone, Mauro Montuori, Mazin Ali, Melody García Domínguez, Menna Maria Paola, Micaela Piccoli, Michela Campanelli, Michele De Rosa, Michele Manigrasso, Michele Maruccia, Michele Torre, Michele Zuolo, Miguel Pera, Mihiri Weerasekera, Mikel Prieto, Min Myat Thway, Mohamed Shaat, Mohammad Azfar, Mostafa Shalaby, Muhammad Asif Raza, Muhammad Umar Younis, Muhammed Elhadi, Mujahid Ali, Musab AlThomali, Nadiah Al Amri, Nagendra Dudi-Venkata, Nahar Alselaim, Neil Smart, Nelson Trelles, Nicolò Falco, Niccolo Petrucciani, Nicola Antonacci, Nicola Cillara, Nicolae Gica, Nicolò Pecorelli, Nicolò Tamini, Nikolaos Machairas, Nura Feituri, Nuria Ortega Torrecilla, Octavio Avila Mercado, Ohood AlAamer, Oktay Irkorucu, Omar Alsherif, Oreste Claudio Buonomo, Orestes Valles-Guerra, Orestis Ioannidis, Oscar Isaac Hernández Palmas, Oscar Sanz Guadarrama, Osman Bozbiyik, Pablo Rodrigues, Pamela Milito, Paolo Panaccio, Panagiotis Dorovinis, Paola Prieto, Paolo Baroffio, Patrizia Marsanic, Pawel Ajawin, Peng Soon Koh, Pietro Anoldo, Piotr Major, Qasem Alharthi, Rashid Lui, Riccardo Caruso, Richard Brady, Rishi Rattan, Rishi Singhal, Roberta Angelico, Roberta Maria Isernia, Roberta Tutino, Roberto Peltrini, Rodrigo Tejos, Roosevelt Fajardo, Rossella Elia, Salvador Morales-Conde, Sami Benli, Sara Fuentes, Sara Gortázar de las Casas, Sara Ortiz de Guzmán Aragón, Sara Vertaldi, Selmy Awad, Sergio Gentilli, Sergio Alberto Weckmann Lujan, Serkan Tayar, Shabab Althobaiti, Silvia Di Giovanni, Soliman Ghedan, Sonia Pérez-Bertólez, Sonja Chiappetta, Spiros Delis, Stefano Scaringi, Süleyman Çetinkünar, Stylianos Kykalos, Syed Muhammad Ali, Sylvia Krivan, Tak Lit Derek Fung, Tarik Delko, Tatiana Nicolás López, Tercio De Campos, Teresa Calderón Duque, Teresa Perra, Theodore Liakakos, Theodoros Daskalakis, Tijmen Koëter, Tiku Zalla, Tomás Elosua González, Tommaso Campagnaro, Toure Alpha Oumar, Ugo Grossi, Valentina Sosa, Valentina Testa, Valentina Tomajer, Valeria Andriola, Valeria Tonini, Valerio Celentano, Valerio Voglino, Venkateswara Rao Katta, Víctor Hugo García Orozco, Victor Turrado-Rodriguez, Victor Visag-Castillo, Victoria Graham, Viktor Rachkov, Vincenzo Papagni, Vincenzo Vigorita, Virginia Jiménez Carneros, Vittoria Bellato, Wolf Bechstein, Yuksel Altinel, Zutoia Balciscueta.

## Appendix 2

Dear colleague,

The ACIE Appy Study was performed in 2020, and reported a surge in non-operative management of appendicitis, consistent with other studies that have been published at national and international level. Apart from conservative management being used more frequently, the Appy Study also highlighted some worrisome discrepancies and inequalities in terms of patient and surgical staff protection, perioperative screening and management, and access to resources.

The aim of the ACIE Appy-2 Study is to give an updated view of how patients with acute appendicitis are being managed globally in 2021 at centres that participated in the ACIE Appy Study, in order to define whether a paradigm shift towards non operative management has occurred and if the inequalities between countries have been currently overcome. Respondents from each center will be listed as Co-authors in the final publications.

**Table:**

	*1 Baseline Information*
1	Your email address
2	Name and Surname
3	In which Country are you practicing?
4	In which Continent are you practicing?
5	Name of your hospital
6	Town
	*2. Hospital organisation and screening policies for COVID-19 during 2021*
7	How did your hospital change its organization in relation to COVID-19 pandemic in 2021? •My hospital is exclusively dedicated to COVID-19 patients •My hospital has restricted areas dedicated to COVID-19 patients •My hospital does not treat COVID-19 patients
8	Did you routinely screen patients with appendicitis for SARS-CoV-2 infection before surgery in 2021? •Yes, all patients •Only patients with respiratory symptoms or suspect of SARS-CoV-2 infection •No
9	How did you screen patients in 2021? •Chest X-ray •Chest X-ray and serology •Chest X-ray and PCR •Chest CT scan •Chest CT scan and serology •Chest CT scan and PCR •Serology •PCR •Rapid test •No screening
	*3. Personal protective equipment PPE against COVID-19 in 2021*
10	Which of the following personal protective devices did you use in COVID-19 negative patients in 2021? •No personal protective devices •Face mask (FFP2/FFP3) •N95 face mask •Goggles •Face mask (FFP2/FFP3) and goggles •N95 mask and goggles
11	Which of the following personal protective devices did you use in COVID-19 untested patients in 2021? •No personal protective devices •Face mask (FFP2/FFP3) •N95 Face mask •Goggles •Face mask (FFP2/FFP3) and goggles •N95 mask and goggles
12	Which of the following personal protective devices did you use in COVID-19 positive patients in 2021? •No personal protective devices •Face mask (FFP2/FFP3) •N95 face mask •Goggles •Face mask (FFP2/FFP3) and goggles •N95 mask and goggles
	*4. Personal attitude: management of acute appendicitis in 2021*
13	Personal attitude: Did you change your attitude in the management of uncomplicated acute appendicitis during 2021 due to the COVID-19 pandemic? •Yes •Yes, only in COVID + patients •Yes, only in COVID + and untested patients •No
14	Personal attitude: How did you manage uncomplicated (no abscess) acute appendicitis during 2021? •Non-operative management with antibiotics •Case-by-case decision •Straightforward laparoscopic appendectomy •Straightforward open appendectomy
15	Personal attitude: How did you manage complicated (abscess) acute appendicitis during 2021? •Non-operative management with antibiotics •Non-operative management with antibiotics and percutaneous drainage •Straightforward laparoscopic appendectomy •Straightforward open appendectomy
16	Personal attitude: Did you change your attitude in the management of complicated (abscess) acute appendicitis during 2021 due to the COVID-19 pandemic? •Yes •Only in COVID + patients •No
17	Personal attitude: Did you change the surgical approach (open vs. laparoscopic) to appendectomy due to the COVID-19 pandemic in 2021? •No •Yes, from laparoscopic to open •Yes, from open to laparoscopic
18	Personal attitude: How did you operate on COVID-19 positive patients with appendicitis in 2021? •Always open surgery, personal preference •Laparoscopic surgery without specific devices for protection and smoke evacuation •Laparoscopic surgery with specific devices for protection and smoke evacuation •I would use laparoscopic, but I do not have devices for pneumoperitoneum/smoke evacuation
19	Personal attitude: How did you operate on COVID-19 untested patients with appendicitis in 2021? •Always open surgery, I prefer •Laparoscopic surgery without specific devices for protection and smoke evacuation •Laparoscopic surgery with specific devices for protection and smoke evacuation •I would use laparoscopic, but I do not have devices for pneumoperitoneum/smoke evacuation
20	Personal attitude: If laparoscopic appendectomy was performed in 2021, did you use any filter system? •Yes •Yes, only in COVID-19 positive patients •Yes, only in COVID-19 positive or untested patients •No
21	Personal attitude: If any evacuation system with filters were used in 2021, which type of device did you employ? •Commercially available •Commercially available with filtration connected to a container with water •Commercially available with filtration connected to a sealed container •Homemade •Homemade with filtration connected to a container with water •Homemade with filtration connected to a sealed container
	*5. Acute appendicitis patients presentation during the COVID-19 pandemic in 2021*
22	Did any patient referred for acute appendicitis test positive for SARS-CoV-2 before surgery at your hospital (percentage) in 2021? •0% •1–5% •6–10% •> 10%
23	Did any COVID-19 negative patient referred for acute appendicitis later test positive for SARS-CoV-2 at your hospital (percentage?) in 2021? •0% •1–5% •6–10% •> 10%
24	How many patients with acute appendicitis have been referred to your hospital during the last month? •< 5 •5–10 •11–20 •> 20
25	In percentage, in how many patients with uncomplicated acute appendicitis (no abscess) a non-operative management with antibiotics is currently used at your hospital? •≤ 25% •26–50% •51–75% •76–100%
26	In percentage, how many patients with uncomplicated acute appendicitis (no abscess) treated conservatively with antibiotics are currently sent home and followed-up at the outpatient clinic at your hospital? •≤ 25% •26–50% •51–75% •76–100%
27	In percentage, how many patients with complicated acute appendicitis (with abscess) currently undergo conservative treatment with antibiotics ± percutaneous drainage at your hospital? •≤ 25% •26–50% •51–75% •76–100%
28	In percentage, how many patients with acute appendicitis treated with surgery currently undergo open appendectomy at your hospital? • ≤ 25% •26–50% •51–75% •76–100%
29	In percentage, in how many patients with acute appendicitis treated with surgery did you use an endostapler to secure the appendiceal stump in 2021? •≤ 25% •26–50% •51–75% •76–100%
30	In percentage, in how many patients with acute appendicitis treated with surgery did you use a High-Energy Device to dissect the mesoappendix in 2021? •≤ 25% •26–50% •51–75% •76–100%
	*6. Impact of guidelines and scientific divulgation on the daily clinical practice for patients with acute appendicitis during the COVID-19 pandemic in 2021*
31	Your current surgical practice has been most likely influenced by •A guideline (please, specify which, with information on a publication from it, if available) •A scientific article (please, specify which one)
32	Have you changed your management strategies in patients with acute appendicitis following a specific guideline/scientific article about surgical management during the COVID-19 pandemic? •I would have, but I did not have resources (staff, equipment, etc.) •Only in part, because of resources restrictions •Only in part, because I or my institution did not agree with published recommendations •Yes, completely adhering to published recommendations
33	How many patients with acute appendicitis referred to your hospital during the last month completed the full vaccination against SARS-CoV-2? •≤ 25% •26–50% •51–75% •76–100%
34	How many surgeons are there in your department who have completed the full vaccination against SARS-CoV-2? •≤ 25% •26–50% •51–75% •76–100%
35	Do you implement any individualized risk-prediction model for COVID-19 in your daily clinical practice? •Yes (Describe) •No
36	Did you receive any local and/or national plan from your hospital for the management of COVID-19 suspected/positive patients? •Yes •No
